# Pseudophosphatase MK-STYX Alters Histone Deacetylase 6 Cytoplasmic Localization, Decreases Its Phosphorylation, and Increases Detyrosination of Tubulin

**DOI:** 10.3390/ijms20061455

**Published:** 2019-03-22

**Authors:** Yuming Cao, Dallas A. Banks, Andrew M. Mattei, Alexys T. Riddick, Kirstin M. Reed, Ashley M. Zhang, Emily S. Pickering, Shantá D. Hinton

**Affiliations:** 1 Department of Biology, Integrated Science Center, College of William and Mary, Williamsburg, VA 23185, USA; yuming.cao@umassmed.edu (Y.C.); dbanks13@umd.edu (D.A.B.); ammattei@email.wm.edu (A.M.M.); atriddic@aggies.ncat.edu (A.T.R.); kmreed01@email.wm.edu (K.M.R.); amzhang@email.wm.edu (A.M.Z.); espickering@email.wm.edu (E.S.P.); 2 Department of Cell Biology and Molecular Genetics, University of Maryland, College Park, MD 20742, USA

**Keywords:** pseudophosphatase, MK-STYX (MAPK (mitogen-activated protein kinase) phosphoserine/threonine/tyrosine-binding protein), HDAC6 (histone deacetylase isoform 6), post-translational modification, microtubules

## Abstract

The catalytically inactive mitogen-activated protein (MAP) kinase phosphatase, MK-STYX (MAPK (mitogen-activated protein kinase) phosphoserine/threonine/tyrosine-binding protein) interacts with the stress granule nucleator G3BP-1 (Ras-GAP (GTPase-activating protein) SH3 (Src homology 3) domain-binding protein-1), and decreases stress granule (stalled mRNA) formation. Histone deacetylase isoform 6 (HDAC6) also binds G3BP-1 and serves as a major component of stress granules. The discovery that MK-STYX and HDAC6 both interact with G3BP-1 led us to investigate the effects of MK-STYX on HDAC6 dynamics. In control HEK/293 cells, HDAC6 was cytosolic, as expected, and formed aggregates under conditions of stress. In contrast, in cells overexpressing MK-STYX, HDAC6 was both nuclear and cytosolic and the number of stress-induced aggregates significantly decreased. Immunoblots showed that MK-STYX decreases HDAC6 serine phosphorylation, protein tyrosine phosphorylation, and lysine acetylation. HDAC6 is known to regulate microtubule dynamics to form aggregates. MK-STYX did not affect the organization of microtubules, but did affect their post-translational modification. Tubulin acetylation was increased in the presence of MK-STYX. In addition, the detyrosination of tubulin was significantly increased in the presence of MK-STYX. These findings show that MK-STYX decreases the number of HDAC6-containing aggregates and alters their localization, sustains microtubule acetylation, and increases detyrosination of microtubules, implicating MK-STYX as a signaling molecule in HDAC6 activity.

## 1. Introduction

MK-STYX (MAPK (mitogen-activated protein kinase) phosphoserine/threonine/tyrosine-binding protein) is a catalytically inactive member of the protein tyrosine phosphatase (PTP) superfamily, classified as a pseudophosphatase [1,2,3,4]. In MK-STYX, critical histidine and cysteine residues in its PTP active site signature motif (HCX_5_R), which are essential for phosphatase activity, are replaced by phenylalanine and serine residues (FSTQGISR) [1,2,3]. Despite being catalytically inactive, MK-STYX plays an important regulatory role in a number of cellular pathways, including apoptosis, neurite formation, and stress granule assembly [3,4,5,6,7,8,9,10]. Elucidating the molecular mechanisms by which MK-STYX functions as a signaling molecule is a subject of much interest. For example, MK-STYX has been shown to promote stress-activated mitochondrial-dependent apoptosis and release of cytochrome c, by sequestering an inhibitor of apoptosis, the mitochondrial phosphatase, PTPM1 (PTP localized to the mitochondrion 1) [8,9]. Similarly, MK-STYX binds G3BP-1 (Ras-GTPase activating protein SH3 domain binding protein-1) [3,4,10], a nucleator of stress granules [11], thereby inhibiting stress granule formation. Stress granules are cytoplasmic aggregates of stalled mRNA [11], which accumulate as an immediate protective response to a stressful environment [12]. Interestingly, the inhibitory effects of MK-STYX are independent of the phosphorylation status of G3BP-1 at Ser149, the critical residue that regulates its activity as a stress granule nucleator [4,10,11].

In addition to MK-STYX and G3BP1, there have been numerous other proteins implicated in the stress granule life cycle [13]. Among them is histone deacetylase isoform 6 (HDAC6). HDAC6 is a member of the HDAC superfamily, which, along with the histone acetyltransferase (HAT) superfamily, regulates gene expression and chromatin remodeling [14]. Important antagonistic actions of HATs and HDACs are accomplished by post-translational modification of histones [15,16], which tightly pack DNA into chromatin. In particular, HATs acetylate lysine residues of histones, loosening the packed DNA and making it available for transcription [16]. HDACs remove the acetyl (CH_3_CO_2_) group from histones, repressing gene expression [17,18]. HATs and HDACs are primarily localized in the nucleus [19]; however, there are non-canonical classes of HDACs that are localized in the cytoplasm [14,19]. The substrates of cytoplasmic HDACs are non-histone proteins such as heat shock proteins, tubulin, and cortactin [20]; they regulate various important cellular processes such as cilia formation, ubiquitination, and autophagy [14,21,22,23]. HDAC6, the prototypical cytoplasmic HDAC [24], is involved in a variety of cellular processes including cytoskeleton-associated stress responses and cell signaling [14,20,23].

HDAC6 contains two catalytic domains, DD1 and DD2, both of which are indispensable for its deacetylase activity. A dynein-binding domain (DMB) separates the two catalytic domains and is required for the recruitment of HDAC6 onto the microtubule motor protein dynein [19,25,26]. Within the HDAC6 N-terminus, there is a nuclear export signal (NES) and a SE14 domain, which ensure the stable anchoring of HDAC6 in the cytoplasm. The C-terminus of HDAC6 contains a cysteine and histidine-rich ZnF-UBP (zinc-finger ubiquitin binding protein) domain that binds mono- and poly-ubiquitin chains with high affinity [14]. In addition, this domain plays a critical role in the function of HDAC6 in the cellular stress response pathway [22,27]. HEK/293 (human embryonic kidney cells) cells treated with HDAC6 catalytic inhibitors, such as trichostatin A (TSA), have fewer or no HDAC6-positive stress granules [28], indicating that the deacetylase activity of HDAC6 regulates stress granule formation. Furthermore, HDAC6 binds the stress granule nucleator G3BP-1, acting as a crucial component in stress granule formation [28]. Immunoprecipitation assays showed that an HDAC6-G3BP-1 complex is dependent on the catalytic domain of HDAC6 and the acidic-rich domain of G3BP-1 [28]. Stress induces dephosphorylation of G3BP-1 at phosphoserine 149, which resides in the acidic-rich domain, resulting in stress granule assembly [29]. Intriguingly, HDAC6 binds with higher affinity to dephosphorylated G3BP-1 than phosphorylated G3BP-1 [28].

Given that both HDAC6 and MK-STYX interact with G3BP-1, we sought to ascertain whether MK-STYX has an impact on the dynamics of HDAC6. Here, we demonstrate that HDAC6 is cytosolic and nuclear in the presence of MK-STYX, instead of solely cytosolic. We also show that MK-STYX inhibits HDAC6 aggregate formation under stress conditions and decreases HDAC6 phosphorylation at Ser22, tyrosine phosphorylation, and lysine acetylation. Microtubule organization was not disrupted by MK-STYX. However, when cells were treated with nocodazole, which normally depolymerizes microtubules and decreases the acetylation and detyrosination of tubulin in certain cells [30,31,32], MK-STYX induced an increase in microtubule detyrosination.

## 2. Results

### 2.1. MK-STYX Causes HDAC6 Localization to Become Whole Cell (Nuclear and Cytosolic) and Decreases the Number of HDAC6 Aggregates

**Figure 1:**
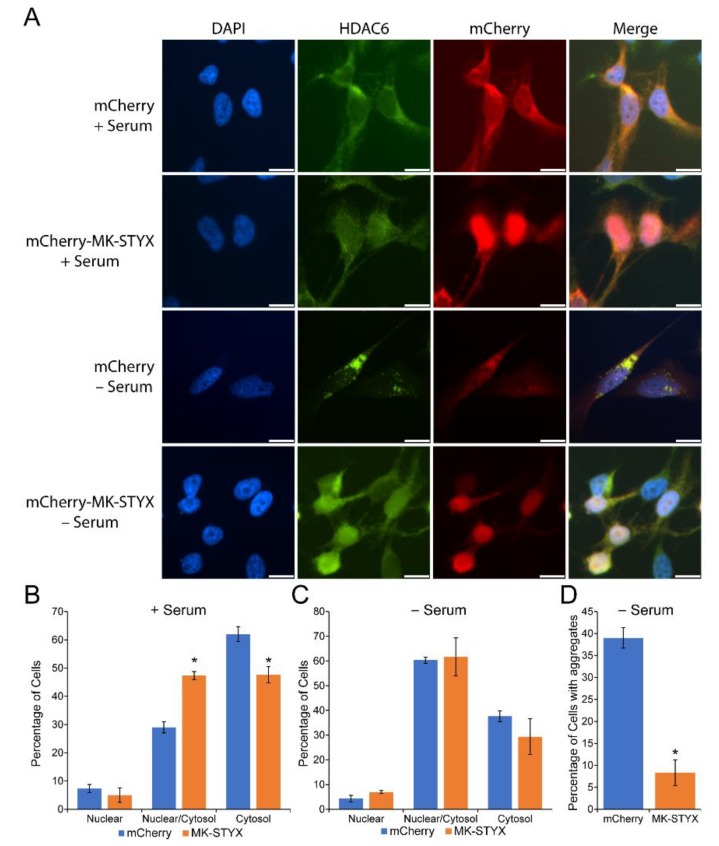
MAPK (mitogen-activated protein kinase) phosphoserine/threonine/tyrosine-binding protein (MK-STYX) causes histone deacetylase isoform 6 (HDAC6) to become partly nuclear and decreases HDAC6 aggregates. (**A**) Representative examples of the subcellular distribution of endogenous HDAC6, detected with an anti-HDAC6 antibody (anti-HDAC6) and anti-rabbit conjugated to FITC, in HEK/293 cells transfected with mCherry (control vector) or mCherry-MK-STYX. We scored the localization of HDAC6 as cytosolic or whole cell (cytosolic and nuclear) in the (**B**) presence or (**C**) absence of serum, in HEK/293 cells overexpressing mCherry-MK-STYX or mCherry control plasmid. (**D**) We scored the number of cells with HDAC6 aggregates that formed in the absence of serum. Paired *t*-test statistical analysis was performed; the error bars are ±SEM; * *p* < 0.05 for mCherry compared to mCherry-MK-STYX. Scale bar, 10 μm. The quantified data is the cumulative output of *n* = 3 independent biological experiments. Taking all fields of view into account, cell density was comparable for cells expressing mCherry and mCherry-MK-STYX. Representative images were chosen to illustrate the subcellular distribution of HDAC6.

HDACs are grouped into four classes, I, II, IV (Zn^+^ dependent proteases), and III (sirtuins; NAD^+^ mechanism) according to their structural homologies [33,34]. Class II consists of HDAC6, which is unique because it is a cytosolic protein with a nuclear export signal (NES) and cytoplasmic anchoring domain [20,26]. To determine whether MK-STYX affects the subcellular localization of HDAC6, HEK/293 cells were transfected with expression plasmids for mCherry (vehicle control) or mCherry-MK-STYX. Using immunofluorescence microscopy, we examined the localization of endogenous HDAC6 in the absence or presence of stressful (serum starved) conditions. In control cells overexpressing mCherry under non-stressful conditions, HDAC6 was cytosolic, as expected (Figure 1A,B). However, HDAC6 was both cytosolic and nuclear in the presence of mCherry-MK-STYX under non-stressful and stressful conditions (Figure 1A–C). MK-STYX significantly decreased the cytosolic localization of HDAC6 (Figure 1B); 62% of control cells showed HDAC6 in the cytosol, whereas 47.7% of cells showed a whole cell distribution of HDAC6 in the presence of mCherry-MK-STYX (Figure 1B; *p* < 0.05). HDAC6 shifted towards a whole cell distribution under stressful conditions (serum starvation) (Figure 1C). However, serum starvation did not significantly affect the subcellular localization of HDAC6 between control and MK-STYX-expressing cells (Figure 1C); HDAC6 was localized throughout the cell in both control cells and cells overexpressing mCherry-MK-STYX (Figure 1A,C). Furthermore, HDAC6 formed aggregates in 39% of cells under stress conditions (Figure 1A,D). However, MK-STYX significantly decreased the number of cells with these aggregates (Figure 1A,D); only 8.3% of MK-STYX-expressing cells formed HDAC aggregates (pairwise *t*-test; *p* < 0.05).

**Figure 2:**
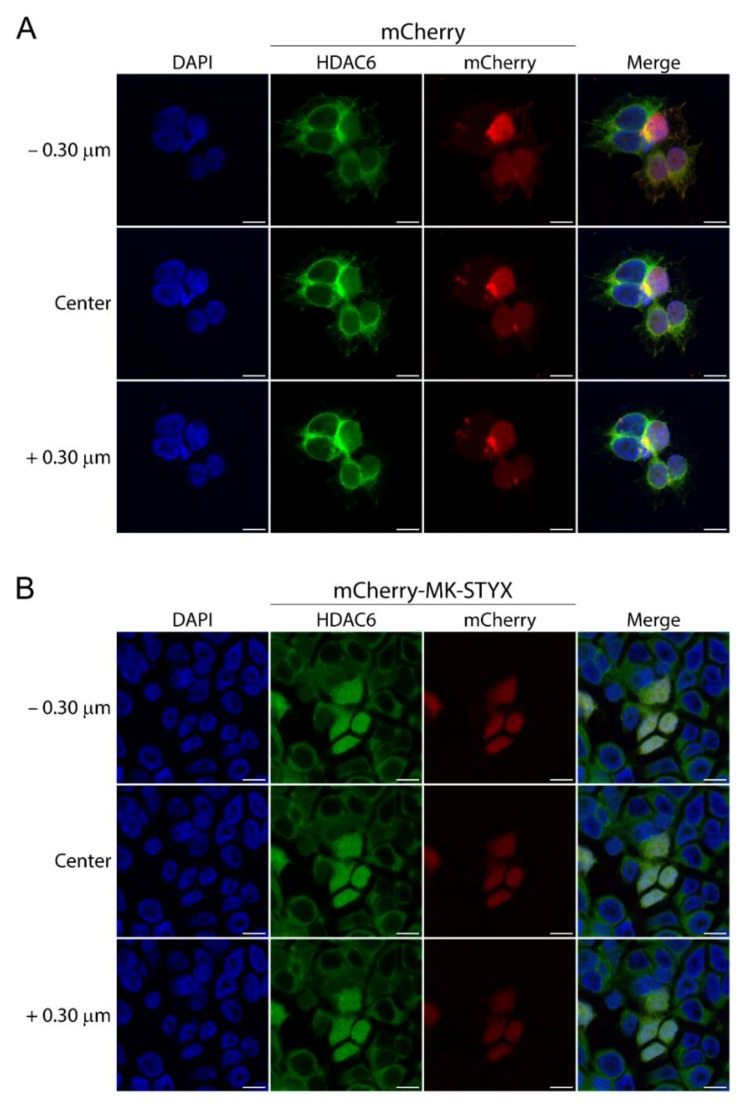
Confocal microscopy validates that the subcellular distribution of HDAC6 is altered in the presence of MK-STYX. (**A**) Representative examples of the subcellular distribution of endogenous HDAC6, detected with an anti-HDAC6 antibody (anti-HDAC6) and anti-rabbit conjugated to FITC in HEK/293 cells, cultured in the presence of serum and transfected with mCherry (control vector) or (**B**) mCherry-MK-STYX. Z-stacks at 0.15 μm were performed by confocal microcopy, starting below the center of the cells. Representative images are shown above (−0.30 μm) and below (+0.30 μm) the center image. Scale bar, 10 μm. Taking all fields of view into account, cell density was comparable for cells expressing mCherry and mCherry-MK-STYX. Representative images were chosen to illustrate the subcellular distribution of HDAC6.

To confirm at higher resolution that HDAC6 was indeed cytosolic and nuclear in cells expressing mCherry-MK-STYX, cells were analyzed by confocal microscopy (Figure 2). Z-stack imaging revealed that HDAC6 is cytosolic in control cells expressing mCherry (Figure 2A), whereas in cells expressing mCherry-MK-SYTX, HDAC6 localizes both in the cytosol and nucleus (Figure 2B). In addition, HDAC6 was also solely cytosolic in cells that did not express mCherry-MK-STYX within the same population of transfected cells (Figure 2B). These Z-stack confocal images validate that MK-STYX shifts the subcellular localization of HDAC6 from cytosolic to whole cell (cytosolic and nuclear).

### 2.2. MK-STYX Alters the Post-Translational Modifications of HDAC6

**Figure 3:**
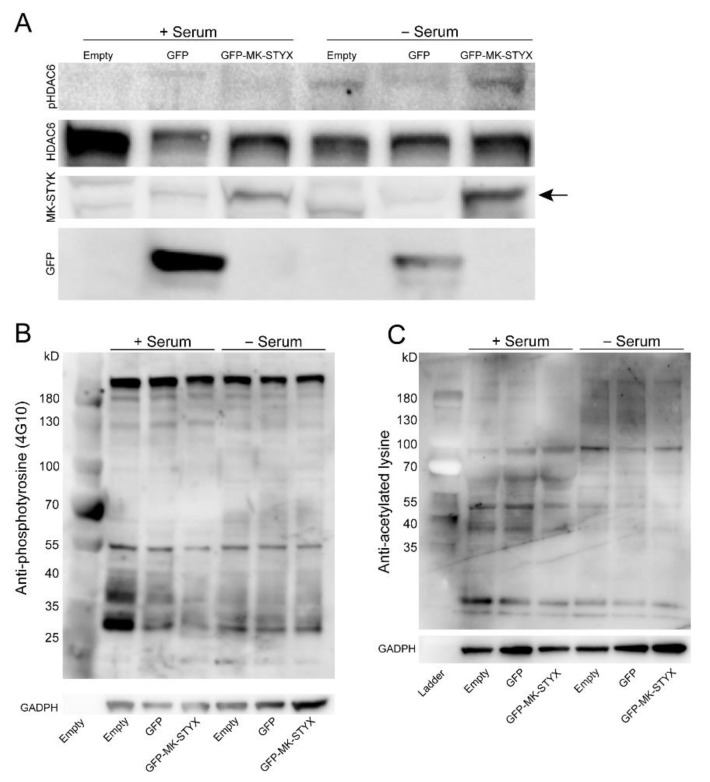
MK-STYX decreases the phosphorylation of HDAC6. (**A**) Twenty-four hours post-transfection cells were serum starved (− serum) or not (+ serum), lysed 24 h thereafter, and immunoblotted. Anti-phospho-HDAC6 antibody showed that MK-STYX decreased HDAC6 phosphorylation in non-serum starved cells relative to the cells overexpressing green fluorescence protein (GFP). However, MK-STYX increased HDAC6 phosphorylation in cells depleted of serum relative to GFP-expressing cells. These blots were stripped and probed for HDAC6 as a loading control. The blot was also stripped and probed with anti-GFP antibody to detect GFP (~27 kDa) or anti-STYXL1 (MK-STYX). Anti-STYXL1 antibody showed overexpressed GFP-MK-STYX (~67 kDa; indicated by the black arrow) relative to GFP (control vector) and non-transfected cells to confirm transfection. Three biologically independent replicate experiments were performed. (**B**) Samples were also used to detect effects of MK-STYX on tyrosine phosphorylation of proteins in the presence and absence of serum with the anti-phosphotyrosine antibody (clone 4G10). Cells expressing GFP-MK-STYX showed decreased phosphorylation relative to the controls in the presence of serum. The blot was stripped and probed with anti-GADPH (glyceraldehyde-3-diphosphatedehydrogenase; 37 kDa) antibody for a loading control. (**C**) Samples were also analyzed for acetylation with anti-lysine acetylation antibody to determine any effects of MK-STYX on acetylated proteins. In the presence or absence of serum, acetylation was decreased in cells expressing MK-STYX relative to control cells. The blot was stripped and probed with anti-GAPDH for a loading control. Three biologically independent replicates were performed.

MK-STYX maintains the characteristic protein tyrosine phosphatase three-dimensional fold and has the ability to bind phosphorylated residues [1,3,35,36]. HDAC6 has multiple phosphorylation sites. Moreover, phosphorylation sites such as Ser22, Tyr570, and Ser458 regulate the deacetylase activity of HDAC6 [37]. To analyze the impact of MK-STYX on the phosphorylation status of HDAC6, we transfected HEK/293 cells with expression plasmids for GFP or GFP-MK-STYX in the absence or presence of serum. Intriguingly, the cells overexpressing MK-STYX showed less HDAC6 phosphorylation at Ser22, compared to control cells expressing GFP (Figure 3A), suggesting that MK-STYX may inhibit a kinase that phosphorylates HDAC6 or promote a phosphatase that dephosphorylates HDAC6 at Ser22. In addition, there was a decrease in tyrosine phosphorylation of proteins in cells overexpressing MK-STYX (Figure 3B). Because acetylation of HDAC6 is also important for HDAC6 function [25,38], we also examined whether MK-STYX alters acetylation. Total protein acetylation was decreased in stressful (absence of serum) conditions in control cells and cells overexpressing MK-STYX. In addition, total protein acetylation was decreased in the presence of serum in cells overexpressing MK-STYX (Figure 3C).

### 2.3. MK-STYX Increases Detyrosination of Tubulin

**Figure 4:**
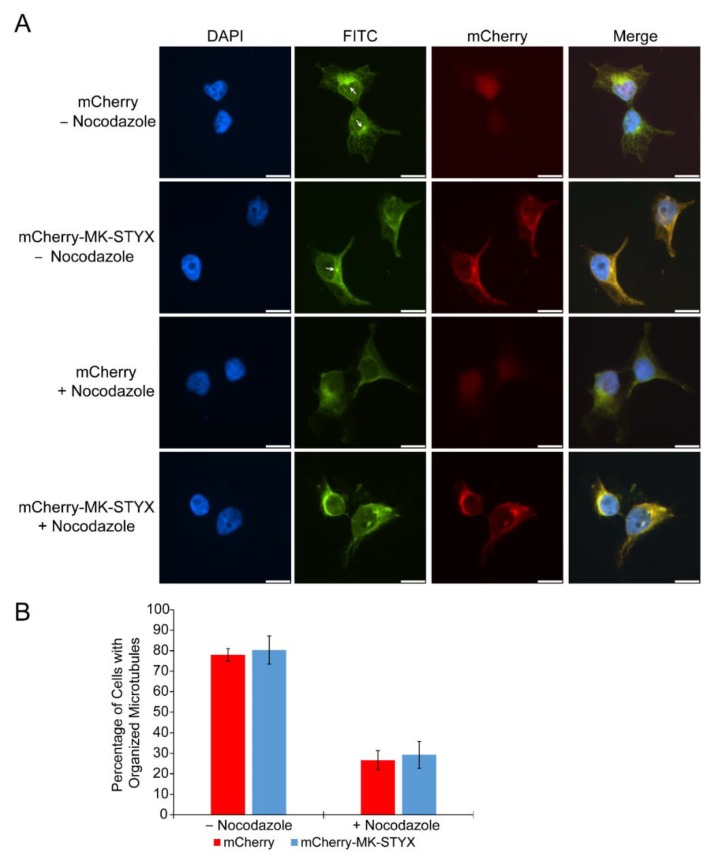
Microtubule organization is stable in the presence of MK-STYX. (**A**) Representative examples of microtubule organization, detected with an anti-tubulin antibody conjugated with FITC, in cells treated or not treated with nocodazole and in cells expressing mCherry or mCherry-MK-STYX, as indicated. (**B**) The number of cells with organized microtubules was scored in the presence or absence of nocodazole. In the absence of nocodazole, microtubules were well-organized in control cells (mCherry) and cells overexpressing MK-STYX, i.e., the microtubules nucleated from the centrosome (white arrow). However, microtubule organization was disrupted (tubulin was diffuse in the cytoplasm) in the presence of nocodazole in both control cells, as expected, and those expressing mCherry-MK-STYX. 100 cells were scored per replicate; three biologically independent replicates were performed. Scale bar, 10 μm. The quantified data is the cumulative output of *n* = 3 independent biological experiments. Cell density was comparable for mCherry and mCherry-MK-STYX. Representative images were chosen to illustrate the subcellular distribution of tubulin.

HDAC6 utilizes the microtubule network to transport ubiquitinated proteins; therefore, it is associated with the microtubule network [39,40,41]. To determine whether MK-STYX affects this microtubule network, we transfected HEK/293 cells with mCherry or mCherry-MK-STYX constructs and observed the organization of microtubules in the presence or absence of nocodazole, which depolymerizes microtubules. MK-STYX did not alter the microtubule network in the absence or presence of nocodazole; microtubules in MK-STYX-overexpressing cells were similar to control cells (Figure 4A). Nocodazole disrupted microtubules (as expected) in both control and MK-STYX-expressing cells (Figure 4B).

**Figure 5:**
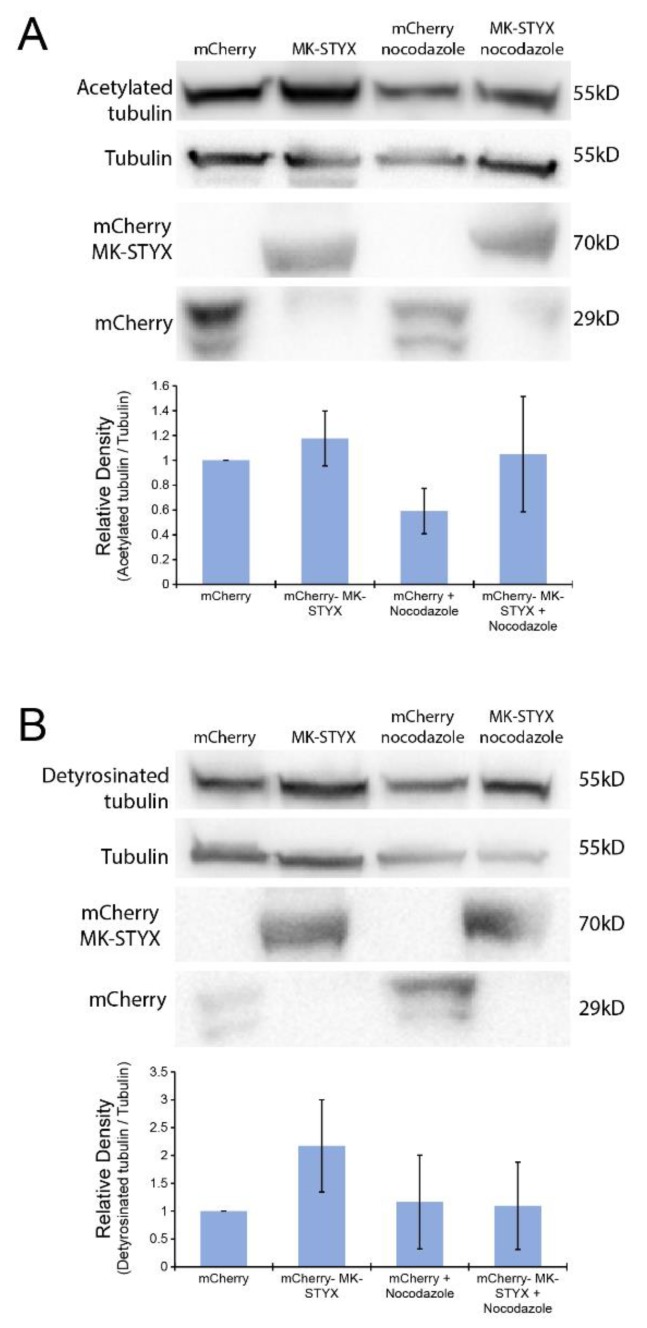
MK-STYX increases acetylated and detyrosinated tubulin. Cells were transfected with expression plasmids for mCherry or mCherry-MK-STYX. Twenty-four hours post-transfection, cells treated with nocodazole or not were lysed and analyzed by immunoblotting. We examined cells expressing mCherry constructs by fluorescence microscopy to confirm transfection. (**A**) Blots were probed with anti-acetylated tubulin and showed that acetylated tubulin (55 kDa) is significantly decreased (paired *t*-test; *p* < 0.05) in the presence of nocodazole in control cells (mCherry-expressing) relative to cells expressing mCherry-MK-STYX. Cells overexpressing MK-STYX sustain acetylated tubulin in the presence of nocodazole. The blots were stripped and probed for tubulin as the loading control; they were also stripped and probed with anti-mCherry to confirm expression of mCherry (27 kDa) and mCherry-MK-STYX (67 kDa). (**B**) Lysates were also analyzed for detyrosinated tubulin (55 kDa) by detection with anti-detyrosinated tubulin. A significant increase (paired *t*-test; *p* < 0.05) in detyrosinated tubulin was observed in mCherry-MK-STYX-expressing cells in the absence of nocodazole, whereas detyrosination was decreased in cells expressing mCherry-MK-STYX in the presence of nocodazole. Blots were stripped and probed with anti-tubulin antibody for a loading control, then stripped a second time and probed with anti-mCherry antibody to confirm transfection. The error bars are ±SEM; three biologically independent replicate experiments were performed.

Because HDAC6 interaction with the microtubule network is dependent on the acetylation and detyrosination of microtubules [23], we also tested whether MK-STYX alters these post-translational modifications (Figure 5). The acetylation of tubulin was significantly decreased in control cells expressing mCherry and treated with nocodazole (paired *t*-test; *p* < 0.05) (Figure 5A); however, tubulin acetylation was unchanged in cells overexpressing mCherry-MK-STYX (Figure 5A), suggesting that MK-STYX sustains the acetylation of tubulin. In addition, MK-STYX significantly increased the detyrosination of tubulin in the absence of nocodazole (paired *t*-test; *p* < 0.05) (Figure 5B), whereas the detyrosination of tubulin was decreased in control and MK-STYX-overexpressing cells under nocodazole treatment (Figure 5B).

## 3. Discussion

HDAC6 catalyzes the deacetylation of various proteins such as tubulin and heat shock proteins. As such, it is an essential regulator of acetylation balance, which is critical for maintaining homeostasis [20]. A disruption of this balance may result in the development of human diseases such as cancer, Parkinson’s, and chronic obstructive pulmonary disease [20,21,22,23,42,43], making it an appealing target for drug therapy and further investigation. This report investigated the effects of the pseudophosphatase MK-STYX on HDAC6. Because both HDAC6 and MK-STYX interact with the stress granule nucleator G3BP-1 and they have antagonistic roles in stress granule formation, promotion and inhibition [4,28], it was important to determine whether MK-STYX affects HDAC6 subcellular location and phosphorylation state, as well as the post-translational modification of its substrate, microtubules. We showed that MK-STYX caused a proportion of this cytosolic protein to localize in the nucleus. Furthermore, in stress conditions the number of HDAC6 aggregates significantly decreased in the presence of MK-STYX. Immunoblots showed that MK-STYX also affected the post-translational modification of HDAC6. Phosphorylation of HDAC6 at Ser22 and total protein tyrosine phosphorylation and acetylation decreased in the presence of MK-STYX. Lastly, post-translational modification of microtubules, which are important for the interaction of motor proteins and HDAC6 [23,44], was also affected by MK-STYX. The acetylation of tubulin increased in cells overexpressing MK-STYX that were treated with nocodazole, which depolymerizes microtubules, compared to control cells treated with nocodazole. In the absence of nocodazole, however, the detyrosination of tubulin was significantly increased in the presence of MK-STYX compared to control cells. Taken together, these data illustrate that MK-STYX influences HDAC6 dynamics at multiple levels: Its subcellular localization, its post-translational modification (phosphorylation), and the post-translational modification of its tubulin substrate. Considering that these dynamics of HDAC6 are crucial for its function of regulating cellular homeostasis, these interactions between MK-STYX and HDAC6 provide insight into the role that MK-STYX plays as a signaling molecule in the stress response pathway [4]. To our knowledge, these are the first studies to identify that MK-STYX serves a role in HDAC6 dynamics.

HDAC6 is a unique member of the class II deacetylases that contains two functional catalytic domains [20,45]. Because of its nuclear export signal (NES) and SE14 domain, HDAC6 was originally thought to be exclusively cytoplasmic; however, numerous reports have revealed that HDAC6 is also present in the nucleus [23,38]. Within the nucleus, HDAC serves as a transcription factor for several biological functions such as ubiquitination, autophagy, and cell motility [23,46,47,48,49]. Furthermore, mouse HDAC6 has been shown to undergo nucleocytoplasmic trafficking [23,49]. The subcellular localization of HDAC6 appears to be cell line dependent; in Jurkat cells, it is more nuclear than cytoplasmic, compared to HEK/293 cells, where it is mostly cytoplasmic [23]. Our present study suggests that MK-STYX causes a conformational change to expose a nuclear localization signal (NLS) of HDAC6 that moves it to the nucleus. Prior reports have shown that acetylation of HDAC6 regulates its subcellular localization; acetylation of lysine 40 is important for the retention of HDAC in the cytosol [21,23,38]. Further investigation of how the dynamics (subcellular localization and post-translational modification) of HDAC6 are altered by MK-STYX may provide important insights into the nuclear and cytosolic functions of HDAC6.

HDAC serves as a mediator of various signaling pathways, including signaling by heat shock factor 1, STAT (signal transducers and activator transcription factors), CREB (cyclic AMP responsive element binding protein), Akt, NFκB, and p53 [48,50]. In particular, HDAC6 regulates ubiquitination and autophagy [22,39]. HDAC6 inhibitors and knockout of HDAC6 have been shown to prevent autophagy [39] and HDAC6 localizes to ubiquitinated organelles such as the mitochondria [51]. Furthermore, HDAC6 is known to bind to monoubiquitinated and polyubiquitinated proteins with high affinity [50]. In the present study, the disappearance of HDAC6 aggregates under stress conditions suggests that MK-STYX has a role in the proteasome pathway. HDAC6 forms aggregates such as aggresomes [52] and stress granules [28,53] under stress conditions [53]. Moreover, HDAC6 is a modulator of lytic granules [54]. Although MK-STYX does not localize to stress granules, it does localize to aggresomes (unpublished data), indicating that MK-STYX may be a modulator of various types of cytosolic granules.

HDAC6 is known to modulate various cytosolic granules through microtubules and motor proteins [52,53]. Microtubules are important structural proteins that provide mechanical function for cells. They are composed of α β-tubulin isoforms, which display a chemical diversity of post-translational modifications such as acetylation, detyrosination, glycylation, phosphorylation, and glutamylation [44]. These modifications of tubulin regulate its properties and recognition by various effectors [44]. HDAC6 mediates the acetylation of the ubiquitin ligase TRIM50 [55] and microtubules [38]. Furthermore, acetylation of HDAC6 prevents it from deacetylating tubulin and promoting HDAC6 import into the nucleus [38]. Prior studies have shown that nocodazole decreases tubulin acetylation [30,31], however, it is important to note that modifications of tubulin are very dependent on the cell line used; post-translational modifications may increase, decrease, or remain unaltered in various cells [30]. In the present study, we showed that acetylation of microtubules significantly decreased in control cells treated with nocodazole. Intriguingly, α-tubulin acetylation was sustained in the presence of MK-STYX, suggesting that MK-STYX inhibits the function of HDAC6. However, the nuclear localization of HDAC6 in the presence of MK-STYX that we observed contradicts the notion of an inactive HDAC6. Prior reports have concluded that when the B subunit of HDAC6 is acetylated, it is unable to deacetylate tubulin and is solely cytosolic [23,38]. Therefore, the acetylation of HDAC6 in the presence of MK-STYX may be at other sites that do not hinder the NLS, but only hinder deacetylase activity. Alternatively, the acetylation sites could be the same and MK-STYX could alter the localization of HDAC6 through other mechanisms. Here, we showed that MK-STYX also increases the detyrosination of α-tubulin, which is important for processes such as wound healing, tubulin stability, and chromosomal direction [44,56].

MK-STYX impacts the dynamics of HDAC6, through altering the subcellular localization and post-translational modification of HDAC6, and the post-translational modification of microtubules, a substrate of HDAC6. These data establish a strong link for the role of MK-STYX in HDAC6 signaling. Further, results support MK-STYX as a regulator in the stress response pathway, while exposing a possible role in the ubiquitin proteasome system. HDAC6 has been previously linked to other phosphatase family members; for example, HDAC6 and protein phosphatase 1 (PP1) form a complex [49,57,58] and HDAC inhibitors disrupt the complex [49]. As another example, Shp2 (non-receptor PTP; encoded by the *Ptpn11* gene) disrupts microtubule regulation by cooperating with HDAC6 to reduce the acetylation and stability of microtubules [59]. Shp2 downregulates RhoA-Dia signaling, resulting in HDAC6-mediated reduction of acetylated microtubules and ERK hyperactivation [59]. HDAC6 has also been linked to the tumor suppressor PTEN (tensin homology phosphatase); combined treatment with celecoxib (cyclooxygenase-2 inhibitor) and an HDAC6 inhibitor activates the PTEN/AKT pathway [15]. The exact mechanism of how MK-STYX regulates HDAC6 signaling requires further study; however, our data highlight the important role that MK-STYX has in this pathway. Moreover, our data corroborate and provide more depth to our previous reports demonstrating the role of MK-STYX in neurite formation [4,5,6]. Acetylation, tyrosination, and detyrosination are important post-translational modifications of microtubules, which have roles in growth cone formation, axon formation, and platelet marginal bands [44]. Our previous reports show that MK-STYX decreases RhoA activation [7] and increases growth cones [6]. Intriguingly, HDAC6 has a role in RhoA-Dia signaling [59]. Here, we demonstrate that MK-STYX modulates the dynamics of HDAC6, providing more insight and depth to our understanding of MK-STYX as a key signaling molecule in diverse cellular pathways. MK-STYX continues to be an important and interesting atypical member of the PTP family to investigate.

## 4. Materials and Methods

### 4.1. Antibodies

The following antibodies were used: Anti-phospho-HDAC6 (pSer22) antibody (Sigma-Aldrich, Burlington, MA, USA; SAB4504190;); anti-HDAC6 (Cell Signaling, Danvers, MA, USA; 7558); anti-GFP antibody (Thermo Fisher, Grand Island, NY, USA c; MA5-15256); anti-phosphotyrosine, clone 4G10 (Millipore; 05-321); anti-acetylated-lysine antibody (Cell Signaling; 9441); anti-STYXL1 antibody (Sigma-Aldrich; S9823); anti-β-tubulin polyclonal antibody (Thermo Fisher; PA1-21153); monoclonal acetylated microtubule antibody (Sigma, Burlington, MA, USA; T7451); anti-tubulin, detyrosinated antibody (Sigma; AB3201); anti-monoclonal anti-mCherry antibody (SAB2702291); monoclonal anti-FLAG M2-FTTC, Clone M2 antibody (Sigma-Aldrich; F4049); monoclonal anti-β-tubulin-FITC (Sigma-Aldrich; F2043); anti-GAPDH (Cell Signaling; 5174).

### 4.2. Cell Culture and Transient Transfection

HEK/293 (ATCC) cells were maintained at 37 °C, 5% CO_2_ in Dulbecco’s Modified Eagle medium (DMEM, Invitrogen, Grand Island, NY, USA) supplemented with 10% fetal bovine serum (FBS). Transfections were performed using Lipofectamine 2000 Reagent (Invitrogen); cells were transfected with expression plasmids pMT2, mCherry, pMT2-FLAG-MK-STYX-FLAG, or mCherry-MK-STYX. Cells were either not stressed or stressed by depletion of serum, or treated with nocodazole for the subsequent experiments, and analyzed by fluorescence microscopy or immunoblotting. When serum starvation experiments were required, cells were maintained in DMEM supplemented with 0.1 % FBS for 8–12 h.

### 4.3. Transient Transfection and Cell Imaging

For immunofluorescence assays, HEK/293 cells were grown to 80–90% confluence and 2×10^5^ cells were plated onto lysine treated coverslips in 6-well dishes (Nunc, Grand Island, NY, USA ). Twelve to eighteen hours post-plating, cells at 40–60% confluence were transfected with 2 μg of mCherry or mCherry-MK-STYX expression plasmid DNA and 4 μL of Lipofectamine 2000 Reagent (Invitrogen) per well, according to the manufacturer’s protocol. The medium was replaced 5 h after transfection. Twenty-four hours post-transfection, cells were serum-starved with DMEM supplemented with 0.1% FBS serum for 8–12, washed with PBS and fixed with 3.7% formaldehyde. The coverslips were mounted to a slide using GelMount containing 4′,6-diamidino-2′-phenylinodole dihydrochloride (DAPI, Sigma) (0.5 mg/mL).

For experiments examining the effect of MK-STYX on HDAC6 subcellular localization, cells were stained with anti-HDAC6 antibody (1:250) dilution (Cell Signaling) for 1 h, then probed with anti-rabbit conjugated Cy3 antibody (1:250) (Cell Signaling). To visualize whether MK-STYX disrupted the microtubules, cells were transfected with mCherry or mCherry-MK-STYX. Anti-β-tubulin-FITC (1:250) was used as the marker to determine the organization of microtubules in the presence or absence of nocodazole. Post-staining, the coverslips were mounted to a slide using GelMount containing DAPI.

Cells were scored for localization of HDAC6 (cytoplasmic or whole cell) and microtubule organization or disruption. Samples were scored blind with regard to treatment and were scored independently by at least two different individuals. At least three biologically independent replicate transfections were performed and at least 100 cells were scored per replicate. Counting and image collection were performed on a Nikon ECLIPSE Ti inverted fluorescence microscope. A Nikon A1Rsi confocal microscope Ti-E-PFS (Nikon Inc., Melville, NY, USA) with a 60× oil objective was utilized to obtain Z-stacks of cells. The 488-nm line of krypton-argon laser with a band-pass of 525/50 nm emission filter was used for GFP detection, the 561-nm line with a band-pass emission filter was used for mCherry and the 405-nm line with a band-pass of 450/500 emission filter was used to detect DAPI. Z-stacks of cells were taken at 0.15 μm. NIS-Elements Basic Research software (version 3.10, Nikon, Brighton, MI, USA) was used for image acquisition and primary image processing and Adobe Photoshop™ and Illustrator™ were used for secondary image processing.

### 4.4. Nocodazole Treatment

Twenty-four hours post-transfection, HEK/293 cells were treated with 6 μg/mL of nocodazole for 30 min, then immediately washed with phosphate buffer saline (PBS) and immediately fixed or lysed for immunoblotting analysis. Fixation studies were analyzed for the organization of microtubules. Immunoblot studies were analyzed for whether the post-translational modifications (acetylation and detyrosination) of microtubules were changed.

### 4.5. Immunoblotting

HEK/293 cells were transfected with pMT2, pMT2-FLAG-MK-STYX-FLAG, GFP, mCherry, GFP-MK-STYX, or mCherry-MK-STYX expression plasmids, then lysed and analyzed by Western blotting. Cells were harvested in lysis buffer (50 mM HEPES, pH 7.2, 150 mM NaCl, 10% glycerol, 10 mM NaF, 1 mM Na_3_VO_4_, 1% Nonidet P-40 alternative (Calbiochem, Burlington, MA, USA), and protease inhibitor cocktail tablets (Roche, Branchburg, NJ, USA). Lysates were sonicated, centrifuged at 14,000× *g* for 10 min, and the supernatant protein concentration was determined by NanoDrop quantification. Lysates were resolved by 4%–20% Bis-Tris gels and transferred to PVDF by the eBlot L1 (Genscript, Piscataway, NJ, USA). Chemiluminescence was detected using a BioRad ChemiDoc MP imaging system for immunoblot analysis with anti-phospho-HDAC6 (pSer22); anti-HDAC6; anti-phosphotyrosine, clone 4G10; anti-acetylated-lysine; anti-STYXL1; anti-GAPDH; anti-β-tubulin polyclonal; monoclonal acetylated microtubule; anti-β-tubulin, detyrosinated; anti-GFP; and monoclonal anti-mCherry antibodies, followed by chemiluminescent detection. When warranted, blots were stripped (200 mM glycine, 3.5 mM SDS, 1% Tween 20) and re-probed.

### 4.6. Statistical Analysis

Paired *t*-tests were used to determine the statistical significance of differences between the subcellular localization of HDAC6 in the absence or presence of MK-STYX under non-stressed and stressed conditions with a significance level of *p* < 0.05 (Figure 1). Paired *t*-tests were also used to determine whether MK-STYX disrupted microtubule organization (Figure 3) and to compare the post-translational modification of control cells (expressing mCherry; Takara Bio, Mountain View, CA, USA) with nocodazole treatment and/or cells overexpressing MK-STYX (mCherry-MK-STYX). To compare all samples to each other, analysis of variance (ANOVA) was performed.

## 5. Conclusions

The shared function of MK-STYX and HDAC6 as regulators in the stress granule life cycle led to investigating whether MK-STYX has a role in HDAC6 dynamics. We have revealed that MK-STYX alters the subcellular distribution and phosphorylation of HDAC6 and sustained the acetylation and increased the detyrosination of tubulin, an HDAC6 substrate. These data enhance our understanding of the pseudophosphatase MK-STYX as a signaling regulator, pose new avenues to pursue in exploring its role in various signaling pathways, and serve as the foundation for further investigation of its dynamics with HDAC6.

## Abbreviations

ANOVA	analysis of variance
DMB	dynein binding domain
FITC	fluorescein isothiocyanate
GAPDH	glyceraldehyde-3-diphosphatedehydrogenase
GFP	green fluorescent protein
G3BP-1	Ras-GAP (GTPase-activating protein) SH3 (Src homology 3) domain binding protein-1
HAT	histone acetyltransferase
HDAC6	histone deacetylase isoform 6
MK-STYX	mitogen-activated protein kinase phosphoserine/threonine/tyrosine-binding protein
NES	nuclear export signal
PTPM1	PTP localized to the mitochondrion 1
PTP	protein tyrosine phosphatase
SEM	standard error mean
SHP2	non-receptor PTP; encoded by the *Ptpn11* gene
ZnF-UBP	zinc-finger ubiquitin binding protein
